# Two mycoheterotrophic orchids from Thailand tropical dipterocarpacean forests associate with a broad diversity of ectomycorrhizal fungi

**DOI:** 10.1186/1741-7007-7-51

**Published:** 2009-08-14

**Authors:** Mélanie Roy, Santi Watthana, Anna Stier, Franck Richard, Suyanee Vessabutr, Marc-André Selosse

**Affiliations:** 1 Centre d'Ecologie Fonctionnelle et Evolutive (CNRS, UMR 5175), Equipe Interactions Biotiques, Montpellier, France; 2 Queen Sirikit Botanic Garden, Mae Rim, Chiang Mai, Thailand

## Abstract

**Background:**

Mycoheterotrophic plants are considered to associate very specifically with fungi. Mycoheterotrophic orchids are mostly associated with ectomycorrhizal fungi in temperate regions, or with saprobes or parasites in tropical regions. Although most mycoheterotrophic orchids occur in the tropics, few studies have been devoted to them, and the main conclusions about their specificity have hitherto been drawn from their association with ectomycorrhizal fungi in temperate regions.

**Results:**

We investigated three Asiatic Neottieae species from ectomycorrhizal forests in Thailand. We found that all were associated with ectomycorrhizal fungi, such as Thelephoraceae, Russulaceae and Sebacinales. Based on ^13^C enrichment of their biomass, they probably received their organic carbon from these fungi, as do mycoheterotrophic Neottieae from temperate regions. Moreover, ^13^C enrichment suggested that some nearby green orchids received part of their carbon from fungi too. Nevertheless, two of the three orchids presented a unique feature for mycoheterotrophic plants: they were not specifically associated with a narrow clade of fungi. Some orchid individuals were even associated with up to nine different fungi.

**Conclusion:**

Our results demonstrate that some green and mycoheterotrophic orchids in tropical regions can receive carbon from ectomycorrhizal fungi, and thus from trees. Our results reveal the absence of specificity in two mycoheterotrophic orchid-fungus associations in tropical regions, in contrast to most previous studies of mycoheterotrophic plants, which have been mainly focused on temperate orchids.

## Background

During the last decade, important advances have been made in our understanding of nutrition of achlorophyllous, heterotrophic plants [1]. Beyond the classical plant-parasitic taxa, a strategy called mycoheterotrophy (MH) has been shown in more than 400 species within several plant clades, showing patterns of convergent evolution to heterotrophy [2]. MH plants receive carbon from soil fungi colonising their roots, forming the so-called mycorrhizal symbiosis [3]. MH in adult (above-ground) life phases has evolved repetitively among orchids [4]. Recent advances were made in identifying mycorrhizal fungi of MH plants by molecular methods, thus revealing their ultimate carbon source, the photosynthesised carbon of autotrophic plants associated with the same mycorrhizal fungi in most cases.

PCR amplification and sequencing of the fungal ribosomal DNA from mycorrhizae allowed identification of MH mycorrhizal fungi in more than a dozen MH orchid species [5-9], as well as in several MH species among Ericaceae [10], Gentianaceae and Corsiaceae [11], and Burmanniaceae [12]. All these studies identified a very specific association, that is, of each MH species with fungi from a single genus or even a sub-clade within a genus. Most fungi involved are mycorrhizal partners on other autotrophic plants, forming arbuscular mycorrhizae (AM) [11-13]. As exceptions, some tropical orchids associate with saprobic fungi [14-17], but are often specific too. Aside from these tropical exceptions, the fungal associates of most MH plants suggest that a carbon flow from surrounding autotrophic plants to the MH plants, *via *the shared mycorrhizal fungus, is likely to occur.

For temperate MH plants, the stable isotope composition of MH plants supports nutrition on ectomycorrhizal (ECM) fungi. Natural abundances in ^13^C and ^15^N are major tools in ecology to detect the food source of an organism [18,19]. Most organisms have a ^13^C abundance similar to their food source, and indeed MH plants have similar or slightly higher ^13^C abundances than associated fungi [20,21]. As an exception, however, ECM fungi are richer in ^13^C than autotrophic plants [22]. Although the reasons for this fractionation are unclear [23], it entails a difference in ^13^C abundance between autotrophic and MH plants [24]. ^15^N accumulates along food chains, due to a fractionation at each trophic level [19], and its abundance usually increases in the order autotrophic plants < ECM fungi ≤ MH plants [20,24]. Moreover, ^14^C labelling experiments have provided direct evidence that MH orchid and Ericaceae receive assimilates from surrounding trees through shared mycorrhizal fungi [25,26].

Current investigations are strongly biased toward MH plants from temperate regions. For example, with the exception of a recent study [16,17], few N and C isotopic analyses have been performed on tropical MH plants. The locations of the laboratories involved, and perhaps the Convention on International Trade in Endangered Species of Wild Fauna and Flora [27], may have limited research on MH species in tropical regions. However, dense cover in tropical forests, which select for light-independent nutrition, provides a useful opportunity to study MH plants. Indeed, among the *ca*. 200 MH orchids, more than 90% occur in tropical regions, including a diversity hot spot in tropical Asia where 120 species grow [4,28]. There have been recent investigations on mycorrhizae from tropical orchids, but they exclusively focused on green, epiphytic species (see, for example, [29,30]). They revealed more or less specific associations with the fungal clades found in autotrophic temperate orchids, the 'rhizoctonias' [31]. This group of otherwise parasitic and saprobic fungi encompasses Ceratobasidiales, Tulasnellales and Sebacinales (from clade B *sensu *Weiss et al. [32]), and is absent from MH species

Our aim was to investigate the identity of the fungal partners and specificity of the association of tropical Asiatic MH orchids, and to compare the putative origin of their carbon with that of temperate MH orchids. In this study, two important factors were taken into account. First, ECM fungi are absent from some tropical forests [3]. We thus focused on tropical Asiatic forests that are dominated by ECM Fagaceae and Dipterocarpaceae tree species [3,33]. Here, as in temperate forests, AM, ECM, and various saprobic fungi are available, as well as rhizoctonias associated with green orchids [34]. Second, we focused on MH species from a clade already studied in temperate regions to control for differences resulting from the orchids' phylogenetic position. The Neottieae, in which MH species arose several times [35,36], are well studied in temperate regions, where they reveal specific associations with ECM fungal clades: Thelephoraceae in *Cephalanthera austinae *[37], Russulaceae in *Limodorum abortivum *[38], and Sebacinales in *Neottia nidus-avis *[25,39].

**Figure 1:**
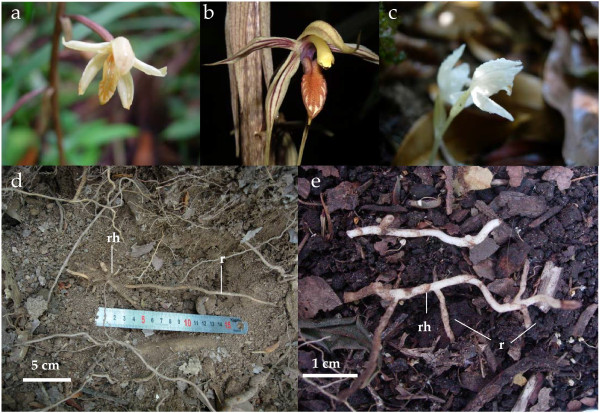
**The three mycoheterotrophic orchid species**. The three mycoheterotrophic orchid species under study, *A. montana ***(a)**, *A. caudata ***(b)**, and *C. exigua ***(c)**, with closer views of the underground parts of *A. montana *roots **(d) **and *C. exigua ***(e)**. Abbreviations: r, root; rh, rhizome.

The tropical Asiatic Neottieae tribe encompasses 33 MH species from the enigmatic genus *Aphyllorchis *[35], and thus represents one of the most diversified MH genera. The position of *Aphyllorchis *among the Neottieae is still not supported by molecular data [35], and even its monophyly is questioned [40]. In this study, we focused on three MH species occurring in ECM forests from Thailand, namely *Aphyllorchis montana*, *A. caudata *and *Cephalanthera exigua *(Figure 1). Assuming phylogenetic conservatism for the traits under study, and based on temperate species already investigated, we expected them to be specifically associated with narrow ECM clades, and to use tree photosynthates by way of shared ECM fungi. Our aims were, within Thailand forests and for these three species, to test these predictions, that is, (i) to confirm that *Aphyllorchis *belongs to Neottieae; (ii) to identify fungal associates of the three species; (iii) to infer their fungal specificity level; and (iv) to investigate their isotopic content in ^13^C and ^15^N, to infer their carbon source.

## Results

### Phylogenetic position of *Aphyllorchis *spp. and *C. exigua*

**Figure 2:**
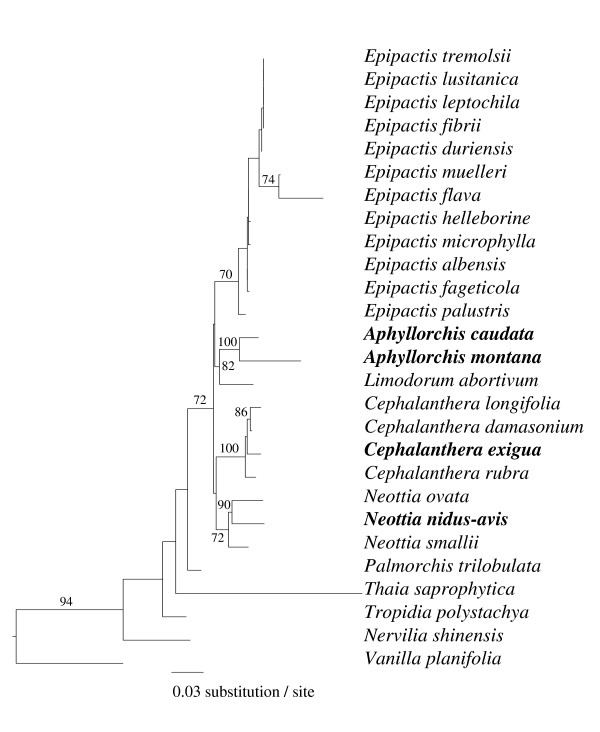
**Phylogenetic tree of the Neottieae tribe**. Phylogenetic tree of the Neottieae tribe showing positions of *A. montana*, *A. caudata *and *C. exigua*; Mycoheterotroph species are in bold. Phylogeny based on a concatenation of ITS, *trnS-G *and *rbcL*, using the maximum likelihood method (general time reversible model). Numbers on branches indicate bootstrap values above 70% (over 1,000 replicates).

Based on three markers (ITS, *trnS-G *and *rbcL*; GB accession numbers FJ454868–FJ454884, Additional file 1), the Neottieae tribe was monophyletic and included the two *Aphyllorchis *under study (Figure 2). *Thaia saprophytica*, a green species from Thailand, had a basal position, but two markers (*rbcL *and *trnS-G*) were not obtained for this species and this limited the support level. Identical topologies at genus level were found, although with lower support levels, when using the three markers separately (data not shown). The genera *Epipactis*, *Listera *and *Cephalanthera *were monophyletic, but this, together with their relative positions, remained weakly supported. The position of *C. exigua *within the genus *Cephalanthera *was well supported, and the two *Aphyllorchis *species clustered together as a well-supported sister clade to the European genus *Limodorum*.

### Molecular identification of root fungi

**Figure 3:**
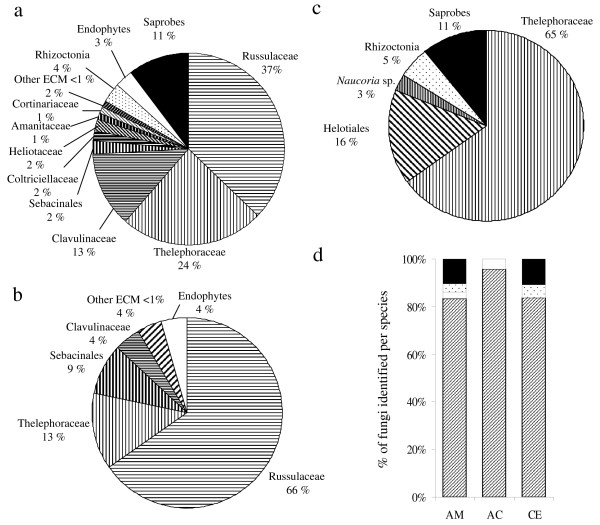
**Description of the community of fungi identified in the three mycoheterotrophic species roots**. Frequency of occurrence of fungal taxa identified in each investigated orchid species grouped on a family/order basis for *A. montana ***(a)**, *A. caudata ***(b)**, and *C. exigua ***(c)**, or grouped by ecology (**(d) **AM: *A. montana*; AC: *A. caudata*; CE: *C. exigua*; ectomycorrhizal taxa are represented by black lines, rhizoctonias by white dots on black background, endophytes by black dots on white background, and saprophytes in white).

**Figure 4:**
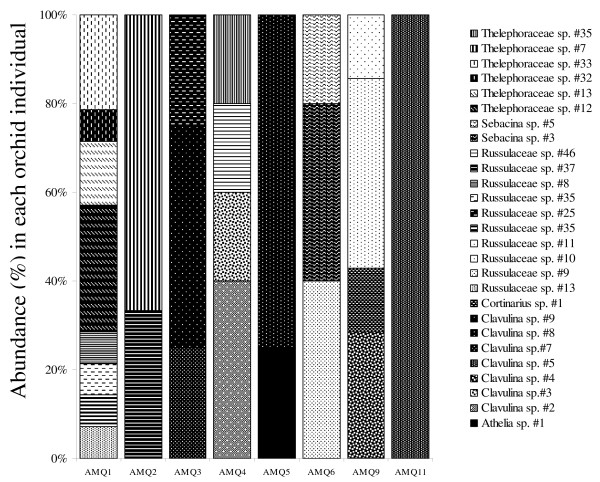
**Differences in ectomycorrhizal taxa between orchid individuals from a given population**. Diversity and abundance of ectomycorrhizal taxa identified in 10 individuals (AMQ1 to 10) from an *A. montana *population (Queen Sirikit Botanical Garden #1).

**Figure 5:**
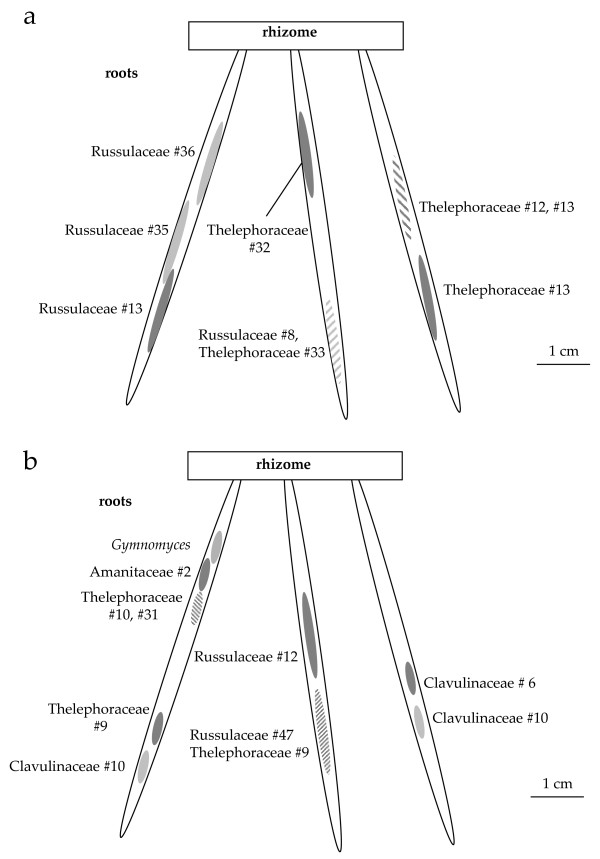
**Distribution of identified fungi on the root system of *A. montana***. Diagram of fungal colonisation on two *A. montana *root systems, on Q1 **(a) **and on D7.1 **(b)**. Numbers correspond to putative species identified (see Additional file 2). Slashed areas display two different fungi, identified on the same 1 to 2 mm-thick root section.

Forty *A. montana *individuals (from seven populations, that is, 288 root samples; Table 1[41] and Additional file 2) produced 220 simple PCR products, representing 135 restriction fragment length polymorphism (RFLP) types, 104 of which were successfully sequenced. In addition, we successfully cloned eight multiple PCR products that produced 11 different sequences (Additional file 2). BLAST identifications showed that 83% were putatively from ECM fungi, 4% from rhizoctonias, 3% from endophytes and 10% from saprobic fungi (Figure 3a and 3d; Additional file 2). ECM fungi belonged to diverse taxa, mainly Russulaceae, Thelephoraceae and Clavulinaceae. Endophytic fungi and *Thanatephorus *sp., a typical orchid mycorrhizal fungus, were found each from a single sample, on individuals also displaying ECM fungi. Thirteen different saprobes were identified, each occurring only on a single sample, except *Resinicium *sp. and *Malassezia *sp. (two samples each). Saprobes were mainly basidiomycetes (69%) and ascomycetes (23%). In all, 15% of orchid individuals did not reveal any ECM fungus, 45% of the individuals revealed a single ECM sequence (sometimes in addition to saprobes and endophytes), and all remaining individuals associated with two to four ECM fungi (Figure 4), sometimes on the same root (Figure 5). Thus, orchid individuals had diverse partners (up to nine putative ECM species from five different genera in a single individual – AMD7.1, Additional file 2, Figure 5). In seven samples, two different sequences were detected by cloning. In five individuals (12.5%) only, identical fungal sequences were retrieved from different roots.

**Table 1:** Description and location of sampling sites.

Species and sampling site	Geocodes and elevation	Type of forest and dominant trees^α^	No. of orchids sampled
*Aphyllorchis montana*			
Doi Suthep #1	18°48'39" N 98°55'00" E 1053 m	Evergreen forest: *Castanopsis acuminatissima *(F)*, Castanopsis diversifolia *(F)*, Dipterocarpus costatus *(D)*, Manglietia garretti *(Magnoliaceae)*, Carallia brachiata *(Rhizophoraceae).	11
Doi Suthep #2	18°48'24" N 98°55'19" E 950 m	Evergreen forest: *Dipterocarpus costatus *(D)*, Castanopsis diversifolia *(F).	11 (+ isotopes samples) ^β^
Queen Sirikit Botanical Garden #1	18°54'24" N 98°51'48" E 811 m	Dry dipterocarpacean forest: *Dipterocarpus costatus *(D)*, Shorea roxburghii *(D)*, Castanopsis argyrophylla *(F)*, Castanopsis tribuloides *(F)*, Lithocarpus thomsonii *(F), *Diospyros variegate *(Ebenaceae)*, Phoebe lanceolata *(Lauraceae)*, Protium serratum *(Burseraceae).	8
Queen Sirikit Botanical Garden #2	18°53'36" N 98°51'27" E 729 m	Oak forest: *Lithocarpus sootepensis *(F)*, Dipterocarpus costatus *(D)*, Shorea roxburghii *(D).	2
Nam Nao	16°93'48" N 101°33'39" E 700 m	Bamboo forest: *Castanopsis diversifolia *(F)*, Lithocarpus *sp (F).	1
Khao Chamao	12°58'41" N 101°42'05" E 800 m	Dipterocarpacean forest: *Dipterocarpus dyeri *(D).	4
Klong Pla Kaeng	12°56'08" N 101°44'09" E 700 m	Dipterocarpacean forest: *Dipterocarpus dyeri *(D).	2

*Aphyllorchis caudata*			
Doi Suthep #3	18°48'39" N 98°55'00" E 1050 m	Evergreen forest: *Dipterocarpus costatus *(D)*, Castanopsis diversifolia *(F).	11 (+ isotopes samples) ^β^
Doi Inthanon	18°35'25" N 98°29'09" E 1000 m	Evergreen forest: *Castanopsis acuminatissima *(F), *Lithocarpus *sp. (F), *Quercus *sp. (F)*, Schima wallichii *(Theaceae).	2

*Cephalanthera exigua*			
Doi Pee Pan Nam	19°06'05" N 99°20°84"E 2015 m	Evergreen forest: *Castanopsis acuminatissima *(F)*, Castanopsis *sp. (F)*, Gironniera *sp. (Ulmaceae), *Lithocarpus *sp. (F)*, Michelia floribunda *(Magnoliaceae), *Myrica esculenta *(Myricaceae), *Neolitsea *sp. (Lauraceae), *Camellia oleifera *(Theaceae)*, Schima wallishii *(Theaceae), *Syzygium angkae, Syzygium *sp. (Myrtaceae).	9 (+ isotopes samples) ^β^

^α ^F: Fagaceae; D: Dipterocarpaceae.

^β ^For species sampled for isotopic studies, see Figure 6.

Nine *A. caudata *individuals (from two populations, that is, 27 samples; Table 1 and Additional file 2) produced 23 simple PCR products that belonged to 12 RFLP types, nine of which were successfully sequenced (Additional file 2). Four multiple PCR products were cloned and produced eight different sequences (Additional file 2). Apart from an endophyte, found only once (*Hypocrea *sp.; Figure 3b and 3d), all sequences were putatively ECM, and mainly belonged to Russulaceae, Thelephoraceae and Sebacinales (from the ECM-forming clade A, *sensu *Weiss et al. [32]). Among all individuals, 55% displayed a single ECM fungus, whereas 45% displayed two to three ECM fungi. As for *A. montana*, no fungal taxon was shared by all individuals.

Nine *C. exigua *individuals (72 samples) from one population produced 63 simple PCR products belonging to 16 RFLP types that were all sequenced. Putative ECM fungi dominated the fungal community (84%), with some rhizoctonias (5%) and saprobes (11%; Figure 3c and 3d). Thelephoraceae represented 65% of identified fungi, and one (FJ454907) was even found in 16 samples arising from seven individuals. Putative ECM Helotiales and *Naucoria *sp. were found in one sample each, as well as saprobes including *Trichoderma *sp. (8% of all fungi) and other ascomycetes (in one sample each). In all, six out of nine individuals exclusively associated with Thelephoraceae, two displayed two different ECM fungi, with a dominance of Thelephoraceae (+80% of the samples), and one displayed only Helotiales. Thus, Thelephoraceae were the preferred fungal associates of *C. exigua*.

### Molecular identification of *A. montana *fungal pelotons

The identity of fungi colonising mycorrhizal cells was assessed on peloton pools (pools of twelve pelotons from a single root section) from two *A. montana *individuals at Doi Suthep #2 (Table 1 and Additional file 2). On AMD6.1, two pools revealed a Helotiales (FJ454973) already found on the same individual, and four revealed a Russulaceae (FJ454956) already found on other Doi Suthep #2 individuals (Additional file 2). On AMD7.1, two fungi already found on the same individual were recovered, namely a Clavulinaceae (FJ454977; three pools) and a Thelephoracae (FJ454979; one pool), while cloning on another pool revealed a mix of the two previous fungi and a Russulaceae (FJ623066, close to *R. illota *and some Russulaceae already found at Doi Suthep #2, Additional file 2). On both individuals, four pools did not amplify. These data corroborated that (i) several ECM fungi were mycorrhizal on the same individual, even the same root, and (ii) ECM asco- and basidiomycetes were mycorrhizal on *A. montana*.

### Analysis of the fungal community analysis

**Figure 6:**
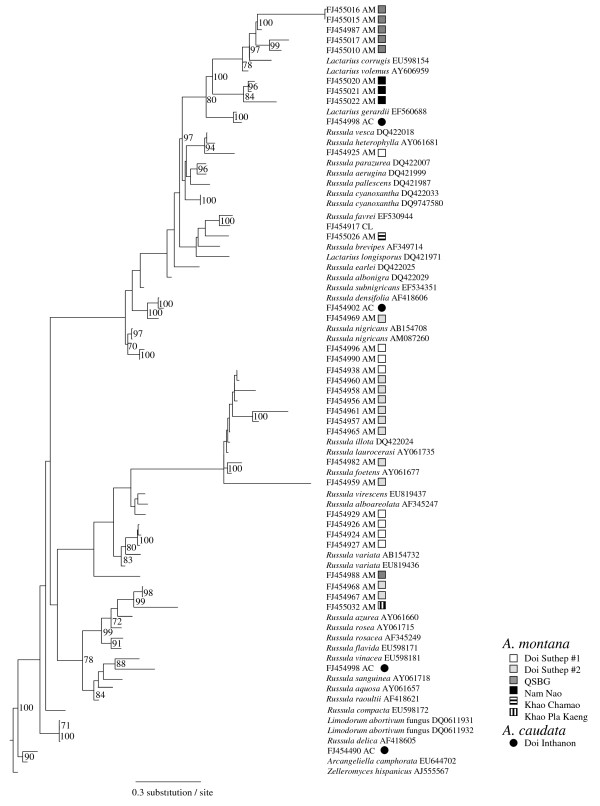
**Over-dispersion of Russulaceae isolated from *A. montana *and *A. caudata***. Unrooted phylogenetic tree placing the Russulaceae identified from *Aphyllorchis montana *(AM) and *A. caudata *(AC). This phylogeny is based on internal transcribed spacer sequences, using maximum likelihood (general time reversible model). Numbers on nodes indicated bootstrap values above 70% (over 10,000 replicates).

Russulaceae, by far the most represented on *A. montana *and *A. caudata *(39.8% of the sequence found, in 33.6% of typed samples), were phylogenetically over-dispersed (Figure 6), further supporting the low specificity of mycorrhizal association. Even fungi identified from the same individual did not cluster together (data not shown), and different Russulaceae species sometimes colonised the same root (Figure 5). Considerable internal transcribed spacer (ITS) variations in Thelephoraceae (also very frequent, 30.0% of the sequence found on *Aphyllorchis *spp. and 63.2% on *C. exigua*) forbade phylogenetic analysis, but sequences were not more similar within than between orchid species (data not shown).

**Figure 7:**
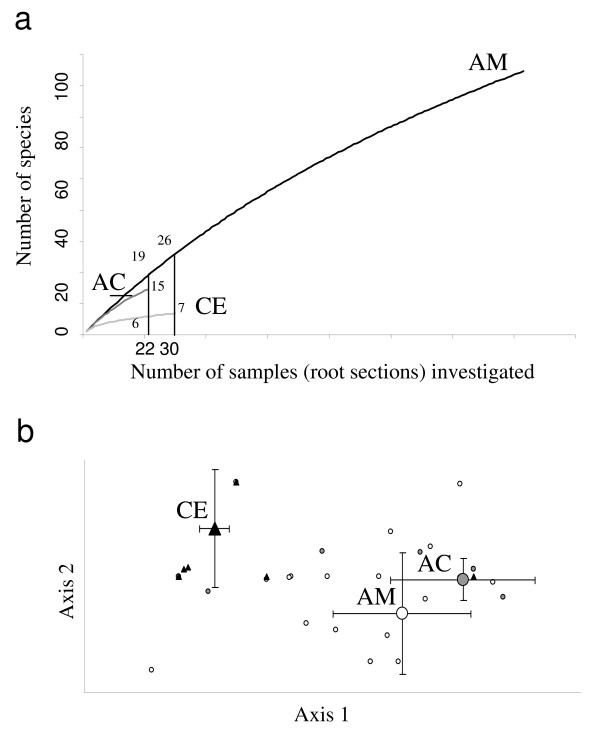
**Differences between the three mycoheterotrophic orchid fungal communities**. Comparison of the fungal communities found on the three orchid species (AM, *A. montana*, AC, *A. caudata *and CE, *C. exigua*). **(a) **Rarefaction curves for ectomycorrhizal fungal species. **(b) **Detrended component analysis of orchid individuals plotted in two dimensions, based on ectomycorrhizal fungal communities (fungal taxa grouped by families; note that most points are superimposed). White circles: *A. montana *individuals; grey circles: *A. caudata*; black triangles: *C. exigua*. Large symbols represent means for each species, with standard deviations.

Using a threshold of < 97% of ITS variation to delineate species, 112 species were recorded in *A. montana*, 23 in *A. caudata *and 31 in *C. exigua*. In all, 94% of these species were represented by a single sequence. Only four species occurred on more than one individual, and were all from the same *A. montana *population (Additional file 2). Rarefaction analyses provide similar trends when (i) considering either all fungi or ECM fungi only; (ii) making the analysis at fungal family or species level; and (iii) pooling all populations or separating them to calculate mean values for each species. In every case, curves for *A. montana *and *A. caudata *were similar (Figure 7a), and higher than for *C. exigua*, so that the low fungal diversity in this species was not a sampling artefact. In detrended component analysis (DCA), no differences in ECM fungal community were found between *A. montana *populations (data not shown) or between the two *Aphyllorchis *species (Figure 7b). In contrast, the *C. exigua *ECM fungal community differed from two *Aphyllorchis *species (*P *< 0.01 for both tests; Figure 7b). Neither the forest type nor the geographical origin had a significant effect (*P *> 0.05; data not shown). Results were unchanged when considering all fungi. Thus, *C. exigua *strongly differed in fungal community structure from the two *Aphyllorchis *species, both quantitatively and qualitatively.

### Stable isotope analyses

**Figure 8:**
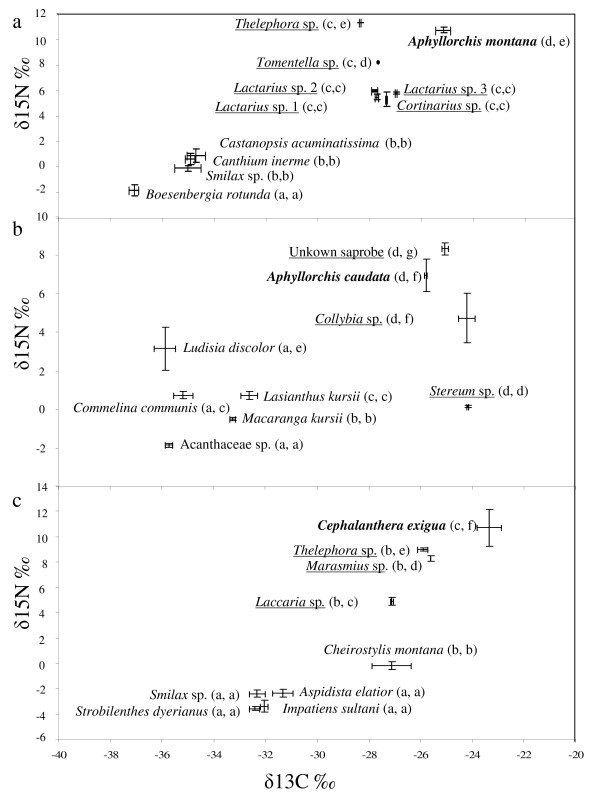
**Isotopic signature of the three mycoheterotrophs studied and other green orchids**. Carbon versus nitrogen stable isotope values (‰) of green plants, mycoheterotrophic plants (names bold) and fungi (names underlined) at **(a) **Doi Suthep #2 (including *A. montana *and various ectomycorrhizal (ECM) fungi), **(b) **Doi Suthep #3 (including *A. caudata *and various saprobic fungi), **(c) **Doi Pee Pan Nam (including *C. exigua*, two ECM fungi and a saprobic *Marasmius*). Letters in brackets denote significant differences between species for both δ^13^C (first letter) and δ^15^N (second letter), according to pairwise Mann-Whitney tests (*P *< 0.01 at least); bars indicate standard deviations.

**Figure 9:**
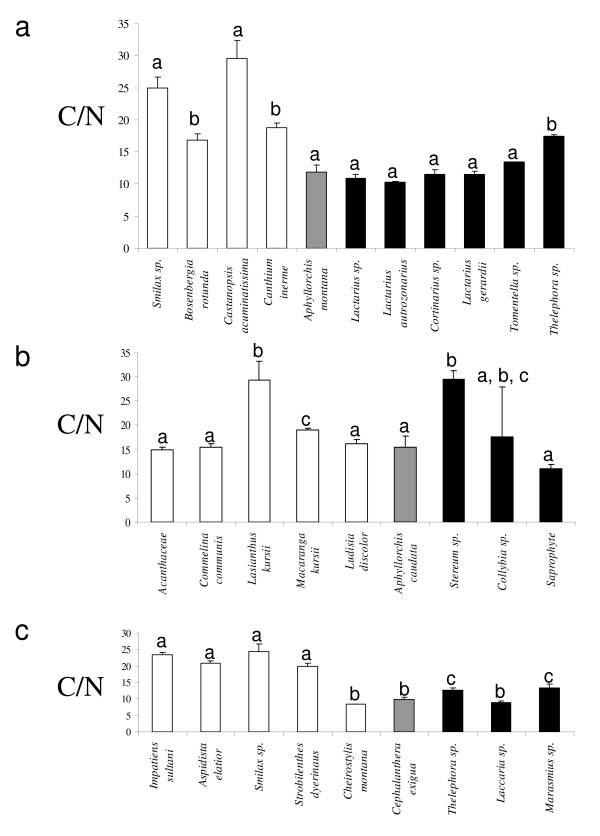
**C/N ratio values of the three mycoheterotrophic orchids and other green orchids **C/N ratio values of green plants, (white bars), mycoheterotrophic plants (grey bars) and fungi (black bars) from three sites: Doi Suthep #2 (**(a)**, including *A. montana *and various ectomycorrhizal (ECM) fungi), Doi Suthep #3 (**(b)**, including *A. caudata*, various saprobic *fungi*), Doi Pee Pan Nam (**(c)**, including *C. exigua*, two ECM fungi and a saprobic *Marasmius*). Letters denote significant differences between species, according to pairwise Mann-Whitney tests (*P *< 0.01 at minimum); bars indicate the standard deviation.

We tested by analyses of natural content in stable isotopes and C/N ratio whether ECM fungi were potential C sources for the MH orchids. At Doi Suthep #2 (Figure 8a), significant differences for both δ^13^C and δ^15^N occurred in the order autotrophic *Boesenbergia rotunda *< other autotrophic plants <*A. montana *≤ ECM fungi (including taxa found on *A. montana *roots, Russulaceae and Thelephoraceae). C/N ratio values were higher for autotrophs than for fungi (12.1 ± 1.2 – mean ± SD) and *A. montana *(11.9 ± 1.2; Figure 9a): the latter two were not significantly different (Mann-Whitney test, *P *= 0.81), but significantly lower than autotrophs (22.6 ± 3.0 on average, *P *< 0.001; *B. rotunda *did not differ from other autotrophs in this respect; Figure 9a). δ^13^C values and variations in δ^15^N and C/N ratio were congruent with a food chain from autotrophs to ECM fungi and *A. montana*.

At Doi Suthep #3, δ^13^C was higher for *A. caudata *than for autotrophic plants but (not significantly) lower than for saprobic fungi. *A. caudata *had δ^15^N intermediate between the different saprobic fungal species, but higher than autotrophs (among them, the orchid *Ludisia discolor *had significantly higher δ^15^N). Unfortunately, no ECM fungi were found, but since they are expected to have lower δ^13^C and higher δ^15^N than saprobes [22], they may well be the orchid's C and N source. Most samples had similar C/N ratio values (Figure 9b). The *A. caudata *ratio (15.5 ± 2.2) did not significantly differ from that of saprobic fungi (15.4 ± 6.9 on average, *P *= 0.65) and was (not significantly) higher than that of autotrophs (18.9 ± 3.2 on average, *P *= 0.38). These results did not reject the hypothesis of a food chain from autotrophs to ECM fungi and *A. caudata*.

At Doi Pee Pan Nam, significant differences for δ^13^C occurred in the order autotrophic non-orchid plants < ECM fungi plus the orchids *Cheirostylis montana *<*C. exigua *(Figure 8c). *C. exigua *and the fungal species had significantly higher δ^15^N than all autotrophic non-orchid plants, and *Cheirostylis montana *showed an intermediate (significantly different) value between these two groups. *C. exigua *C/N ratio (9.8 ± 0.9) did not differ from that of *Cheirostylis montana *(8.3 ± 0.3) and one ECM fungal species (*Laccaria laccata*, *P *= 0.08; Figure 8c), but these values were significantly lower than for other ECM fungi (11.66 ± 1.23, *P *= 0.01), which, in turn, had a significantly lower C/N ratio than non-orchid autotrophic plants (22.2 ± 1.5, *P *= 0.002). These results suggested that (i) *C. exigua *could receive C and N from ECM fungi, and also that (ii) *Cheirostylis montana *may receive part of its C from fungi.

## Discussion

We show for the first time that (i) at least some *Aphyllorchis *belong to the Neottieae tribe; (ii) tropical (Thailand) Neottieae associate with ECM fungi; and (iii) that they are likely to use their ECM fungi (and thus nearby trees) as a C source. This is congruent with what is known from temperate Neottieae species [5,20,21,37], but we provide here the first isotopic evidence that tropical MH orchids associate with ECM fungi. Furthermore, in sharp contrast to the high specificity hitherto found in all investigated MH species [1], and especially MH orchids [9], mycorrhizal associations in the two *Aphyllorchis *species studied here revealed a very low specificity, while *C. exigua *proved to be more specific.

### Tropical MH Neottieae associate with ECM fungi

In temperate regions, ECM fungi consistently associate with roots of Neottieae, both green [36,41-44] and MH [5,25,37,39]. Here, we found that most root fungi had a putative ECM ecology. Although endophytes, saprobes and some rhizoctonias were also found, peloton analysis in *A. montana *only recovered ECM fungi. The mycorrhizal status of putative endophytes and saprobes remains questionable, as in previous studies on *Cephalanthera *spp. [36,41]. ECM fungi are common in Dipterocarpaceae and/or Fagaceae forests of South-East Asia [45,46], and especially of Thailand [47]. The most frequent taxa in this study (Russulaceae, Thelephoraceae) are also the most abundant under Dipterocarpaceae [45,47], where Clavulinaceae and Sebacinales clade A are also known [48]. While temperate Neottieae often associate with taxa forming hypogeous fruit bodies (such as *Tuber *or *Hymenogaster*; [49]), little evidence for this trend was found here (with the possible exception of sequence FJ454490 on *C. exigua*, closely related to the hypogeous *Arcangeliella*, Figure 6). Relatively wet conditions in the investigated forests may explain this, since hypogeous taxa have been shown to be adapted to dry environments [3]. Yet, hypogeous taxa such as Sclerodermataceae exist in Thailand [47]. Moreover, the absence of hypogeous taxa remains difficult to confirm (i) from sequencing data only, and (ii) in a context where the fungal diversity remains poorly explored.

*A. montana *and *A. caudata *harboured a highly diverse ECM community (Additional file 2, Figure 5, Figure 6), very similar for the two species (Figure 6, Figure 7b), dominated by Russulaceae and Thelephoraceae, the latter also dominating on *C. exigua*. Russulaceae are specific associates of the Mediterranean *Limodorum abortivum *[38], a sister species to the genus *Aphyllorchis *(Figure 2); however, the species found here were unrelated to the *R. delica *clade mycorrhizal on *L. abortivum *(Figure 7). *C. exigua *specifically associated with Thelephoraceae, which are specific associates of the related North American MH *C. austiniae *and colonise, although not exclusively, green European and Asiatic *Cephalanthera *spp. [38,41,42,44,50]. The existence of some phylogenetic inertia in fungal preference within Neottieae (or even within the genus *Cephalanthera*) is an appealing possibility that deserves further study, including more species and a more robust phylogeny of this tribe. With the possible reversion of some *Epipactis *sp. [36,42], we confirm here that the Neottieae lost association with the rhizoctonias (the plesiomorphic mycorrhizal feature among orchids) and became associated with ECM fungi irrespective of their global localisation (a *Cephalanthera longifolia *individual from a Myanmar forest also revealed ECM fungi, including Russulaceae – GB accession numbers FJ454917–FJ454919, see Figure 6).

Tropical MH orchids offer considerable diversity in ecology of associated fungi. ECM fungi have already been found in some tropical MH orchids, such as *Lyophyllum shimeji *(in *Erythrorchis ochobiensis *[51]) or ECM Ceratobasidiaceae (in *Chamaegastrodia sikokiana *[52]). Most species associate with non-ECM fungi, that is, parasites [53] or saprobes [14-17,54,55], a fungal ecology never found in temperate MH orchids. This fungal diversity is reflected in the fact the MH *Gastrodia nana *and *Epipogium roseum*, both mycorrhizal with saprobic fungi [14,16,17], also occur in the Thailand forests where this study was carried out (Watthana and Roy, personal observations). In this framework, it is tempting to speculate that other factors, such as contingency or phylogenetic inertia, contribute to the ecology of the fungus in tropical orchids. For Neottieae, the previously mentioned shift from rhizoctonias to ECM fungi [36,42] allowed diversification in ECM forests, not only in temperate regions where such forest dominates, but also in tropical forest harbouring ECM trees. The analysis of mycorrhizal partners in the few Neottieae occurring in tropical America and Africa, as well as in some of the other 33 *Aphyllorchis *species in tropical Asia [35], is now pending, to allow the construction of a global phylogeographic scenario for the Neottieae.

### Tropical MH Neottieae likely receive C from nearby ECM trees

Since ECM fungi almost exclusively receive C from host trees [3], the investigated MH species may indirectly exploit the nearby trees, by way of mycelial links. This was described for temperate species [5,39,56], and corroborated by the high, fungal-like ^13^C and ^15^N in MH plants [20,24]. Here, our isotopic analyses show similar patterns, congruent with C transfer from trees to MH species, via ECM fungi, for tropical sites.

As in temperate ecosystems, δ^13^C were higher for fungi than for autotrophs [57]; unfortunately, the sampling did not allow comparison between saprobic and ECM fungi on each site. Values of δ^13^C tended to be equal or higher for MH orchids as compared with ECM fungi at Doi Suthep #2 (-25.1‰ vs -27.6‰) and Doi Pee Pan Nam (-23.1‰ vs -26.2‰). At Doi Suthep #3, where no ECM fungi were available, saprobic fungi were higher in δ^13^C. Since saprobes usually tend to have higher δ^13^C than ECM fungi [22,57], this site may not contradict the common trend at the two others. Although it is often assumed that δ^13^C are identical in ECM fungi and MH plants [20], some ECM fungi from the same site can be 1 to 2‰ lower in δ^13^C than MH plants [21]. Whether the differences observed here are specific to these tropical models or the result of ECM sampling unrepresentative of the mycorrhizal species is an open question. However, the difference in δ^13^C between MH and autotrophic plants (ranging from 6.8‰ to 9.9‰) was in the range observed in temperate ecosystems (+6.9 ± 1.5‰ [24]), whereas more diverse values were found for MH orchids associated with saprobic fungi (up to +12‰ [16,17]).

Investigated MH orchids tended to have higher δ^15^N and equal to lower C/N ratio values than ECM fungi, as expected between two consecutive levels in a food chain, respectively due to isotopic fractionation for ^15^N [18,20] and the loss of respiratory C [41]. In all, the isotopic data are congruent with a C flow from autotrophs to MH plants by way of shared fungi. Since they do not exclude other scenarios, only a direct labelling of tree photosynthates would definitively assess whether mycelial links between trees and orchids allow a flow to MH plants. In this regard, the putative scenario and C and N data obtained here do not differ from those observed in temperate MH orchids. The existence and roles of common mycorrhizal networks have often been speculated in tropical ecosystems [58], but rigorous demonstration is still lacking: inter-plant C transfers are striking indirect evidence of their existence [26].

### Mixotrophy in tropical orchids

In temperate regions, green plants phylogenetically related to MH plants recover part of their C from their mycorrhizal fungi, especially among orchids [21,38,42,46]. This photosynthetic and partially MH nutrition, also called mixotrophy, is considered as an adaptation to understorey conditions, with low light levels. It can thus be expected in dense tropical forests, but has not yet been demonstrated [2]. Mixotrophy entails ^13^C and ^15^N natural abundances intermediate between those of fully autotrophic and MH plants [21,41,59]. Here, *Cheirostylis montana *at Doi Pee Pan Nam had ^13^C abundance significantly differing from autotrophs and closer to that in ECM fungi and *A. caudata*. Since the ^13^C content (-27.1 ± 1.5‰) is too low for a C4 photosynthetic metabolism [18], mixotrophy is likely to occur. A linear two-sources mixing model [60], with mean δ^13^C values of autotrophs and MH plants as references, suggests that 82% of its C was of fungal origin (significantly different from zero based on 95% confidence intervals).

Mycorrhizal partners of *Cheirostylis montana *have not been investigated yet, but deserve further attention. Indeed, most research on tropical orchid mycorrhizae deals with epiphytic species, and only a few terrestrial species have been studied, using *in vitro *isolation techniques that revealed only rhizoctonia fungi [61]. However, several ECM fungi are difficult or impossible to isolate [6], and therefore molecular approaches are strongly recommended in future attempts to identify fungi of tropical terrestrial orchids. Using such approaches, tropical ecosystems may provide model systems for the examination of mixotrophy in diverse species beyond orchids.

### Absence of mycorrhizal specificity in *Aphyllorchis *spp

The lack of mycorrhizal specificity in *A. montana *and *A. caudata *is unexpected for MH species. Such a low specificity, observed both at population and individual levels (Figure 4), is very unusual among orchids [31], but has been found in some mixotrophic Neottieae in the genera *Cephalanthera *and *Epipactis *[36,41-44]. *Aphyllorchis *species associated with various ECM fungi at the population, individual, root and cell levels (Figure 5), and no obligate or constant partner was identified. In contrast, *C. exigua *associated quite specifically with Thelephoraceae, and suggested that our design did allow detection of specificity. Moreover, rarefaction curves confirmed that for whatever sampling effort, *C. exigua *presented a lower diversity (Figure 7a). Since *Aphyllorchis *fungal communities look like ECM communities from tropical regions [46,62], they may even reflect a random sampling of available ECM fungi by orchids' roots. However, given our limited knowledge of ECM diversity in Thailand forests, we do not know whether there is over- or underrepresentation of ECM fungal taxa colonising nearby trees. Interestingly, Sclerodermataceae, which are common in Thailand dipterocarpacean forests [47], were absent in orchid roots. We thus cannot exclude some limited mycorrhizal preference in *Aphyllorchis *spp. An intriguing consideration is that specificity in green or MH orchids, like *C. exigua *(Figure 1), correlates with short roots (less than 10 cm in length, or even absent; Roy, personal observation), whereas non-specific species, such as *Aphyllorchis *spp., have long roots (up to 50 cm; Figure 1).

*Aphyllorchis *species contrast with the highly specific temperate MH Neottieae studied so far, such as *Neottia nidus-avis *(with Sebacinales [5]) and *C. austinae *(with Thelephoraceae [37]). Ironically, *Aphyllorchis' *closest phylogenetic relative, the mixotrophic *Limodorum abortivum *(Figure 2), specifically associates with the Russulaceae [38]. A strong trend toward specificity is reported in nearly all MH plants [1,6]: individuals are associated with a narrow fungal clade of fungus, and specificity results in local specialisation or even specialisation toward distinct genotypes within populations [7,63]. To the best of our knowledge, the only reported exceptions are (i) two taxa of AM fungi in MH African *Burmannia congesta *and *Sciaphila ledermannii *[64]; (ii) saprobic Basidiomycetes in the Caribbean MH *Wullschlaegelia aphylla *[17], an orchid distantly related to Neottieae; and (iii) the case of another MH orchid, *Erythrorchis cassythoides *[65]. Other tropical MH orchids are specific (for example, [14,52]). Thus, tropical MH orchids exhibit different specificity levels, as reported for tropical green epiphytic orchids [66,67].

There are two caveats to the conclusion of non-specificity. Firstly, we do not know whether all or only some of these fungi are providing C: functional specificity cannot be ruled out. Nevertheless, no constant partner was identified, suggesting that several different fungi can provide C. Secondly, MH nutrition is also taking place at germination and early seedling development in orchids, since seeds have very few reserves: we do not know whether seedlings exhibit fungal specificity. In *Cephalanthera *spp., only a subset of fungi present in adult plants are efficient at this stage [44], and some orchids change or diversify their partners over their lifespan [6]. Indeed, if seedlings also have a large host spectrum, *Aphyllorchis *spp. may not be limited by availability of fungal partners. They are widespread but remain rare, with loose populations (individuals are often separated by a few meters [28], Roy and Watthana, personal observations). Thus, a different specificity in early life stages cannot be excluded in *Aphyllorchis*, and requires further investigation. Several observations of the association during *in situ *germination were obtained in temperate regions, after sowing seeds in mesh bags [31], but this remains to be applied in tropical ecosystems.

### Why is fungal specificity low in tropical MH orchids?

Interestingly, the few non-specific MH plants reported so far occur all in tropical ecosystems [17,64,65]. Although this may be pure coincidence, it may suggest some particular features of MH plants and/or fungal communities in tropical ecosystems. Specificity in biological interactions reveals variable latitudinal patterns, ranging from higher specificity in the tropics (for example, for plant endophytic fungi [68]) to similar or lower specificity (for example, for phytophageous and pollinating insects [69]). Difference between latitudes thus relates more to the functioning of each interaction. However, the raison d'être of MH specificity remains poorly understood in temperate MH species. Two non-excluding models were proposed, namely functional co-adaptation and parasitic co-evolution [70]. Functional co-adaptation states that the mechanism reversing the C flow (which goes from plant to fungus in common mycorrhizae [71]) requires fine plant adaptations to fungal physiology, and that specific adaptations are better than universal ones (functioning with any fungus). However, the many shifts of fungal partners during the evolution of MH lineages [8,10] are not predicted by this model. Parasitic co-evolution assumes that MH plants parasitise their mycorrhizal fungus (and thus 'epiparasitise' on green plants [1]), although there is no direct evidence of detrimental effects [39,70]. In this case, specificity would evolve within an arms race between the fungus and the MH plant: first, epiparasitic plants can only associate with exploitable fungi that are somehow resistant to epiparasitism (non-resistant fungi may not support epiparasites and the association could not be maintained), then both partners may select for adaptations reducing the cost of this association, and such adaptations makes the association more and more specific. As a result, few co-evolved plant-fungus combinations are successful, and evidence for local adaptation in MH populations [10] and co-evolution with fungi [12,63] support this. Our study and a few others [18,64,65] suggest that these mechanisms at least do not apply to tropical MH plants.

We propose a common reason to explain non-specificity in (i) any mixotrophic plants, and (ii) tropical MH orchids. In both cases, the C demand would not be very costly for the fungus. We respectively assume that (i) mixotrophic plants have limited C requirements, because of their photosynthesis, and (ii) due to better tree photosynthesis (higher primary production) in the tropics, tropical ECM fungi receive a greater C flow. In both cases, the C uptake would be relatively negligible, as compared with the C demand of MH plants on temperate ECM fungi. Thus, functional co-adaptation and/or parasitic co-evolution would not apply in tropical regions since avoidance mechanisms are selected only if the cost of avoidance is lower than the cost of interaction [72]. This statement remains speculative, since we know little about the C budget in individual mycelia, and comparative fungal physiology in tropical versus temperate regions. More studies of orchid-fungal diversity in tropical ecosystems are required to support it. Making this assumption, specific MH plants and also some specific temperate mixotrophic plants (such as *Limodorum abortivum *[38]) would simply go beyond a threshold in terms of C loss for the fungus, thus entering the co-adaptation and/or parasitic co-evolution process leading to specificity. Alternative explanations remain possible: heterogeneous environments make generalists fitter than specialists [73,74]. Unfortunately, we do not know the structure and spatial heterogeneity of ECM at our sampling sites, and there is even some evidence that tropical ECM communities are less diverse than temperate ones (K Nara, personal communication).

## Conclusion

All Neottieae examined to date in both temperate and, now, tropical ecosystems have been found to associate with ECM fungi. In most cases, they receive C from ECM mycelial networks linking them to nearby trees, as shown by their isotopic content. During Neottieae evolution, specificity arose repeatedly, but unexpectedly this turns out to be unrelated to full MH nutrition; in spite of several shifts in fungal partners, some phylogenetic inertia may have occurred. The lack of specificity is encountered for a few other tropical MH plants, suggesting that MH and fungal organisms from tropical ecosystems may differ functionally from their temperate analogues. This and the observation of mixotrophy in green orchids calls for more focus on mycorrhizal associations of terrestrial herbaceous plants in the tropics, to know more on the taxonomic position of their fungi and functional diversity (especially in terms of C flow) of their mycorrhizal association.

## Methods

### Model species and sampling sites

*Aphyllorchis montana *Rchb.f., *A. caudata *Rolfe ex Downie and *Cephalanthera exigua *Seidenf. are MH orchids (Figure 1) from South-East Asia that grow in low to high mountain forests [28]. *C. exigua *blooms during the dry season (April), whereas the two *Aphyllorchis *spp. bloom during the rainy season (July to August). All roots were harvested from large populations at the beginning of their flowering period in 2006 and 2007, with the authorisation of the National Council for Research of Thailand. Samples were collected from 10 different sampling sites, separated by 500 m to 1000 km in diverse parts of Thailand (North-West, Central and South-East) with different forest types (evergreen, pine-oak or dry dipterocarpacean forest) – see details and site names in Table 1.

### Sampling for molecular analysis

We harvested three to six independent root fragments (> 3 cm in length) using a protocol that allows plant survival (careful approach to plant roots by digging from one side and, after sampling, refilling of the hole with the same soil without direct rhizome disturbance [8]). We discarded roots specialised in starch accumulation (often occurring in Neottieae [31]) and roots showing infections or symptoms of decay. Within 2 h after harvesting, the remaining roots were carefully washed with water to eliminate soil particles, surface-sterilised using a solution of sodium hypochloride (2% v/v) and Tween 80 (5% w/v) for 10 s, and rinsed three times in sterile distilled water. Roots were then enveloped in paper and stored in silica gel. Next, 1 mm-long sections were sampled every centimetre on the roots, and their colonisation was checked under the microscope using the neighbouring root section (3 to 15 colonised samples were recovered per plant). To identify directly the fungi forming pelotons (intracellular hyphal coils produced by orchid mycorrhizal fungi), pelotons were isolated under a microscope according to Rasmussen [31] on *A. montana *individuals AMD6.1 and AMD7.1 from Doi Suthep #2 (Table 1 and Additional file 2). For 10 root sections per individual, 12 pelotons were recovered and pooled per section (2 × 10 = 20 peloton pools in all).

### Molecular investigations

DNA extraction and PCR amplification of fungal ITS of ribosomal DNA were performed as in Selosse et al. [5] on root fragments and peloton pools. Whenever PCR failed, we tentatively amplified (i) the large mitochondrial ribosomal subunit gene (LrDNA) as in Roy et al. [8], and (ii) the 5' part of the 28S rDNA, using the primers Lr0r and Lr5 as in Roy et al. [8]. Some PCR products with multiple bands were cloned as in Roy et al. [8], and at least five clones per individual were recovered. Before sequencing, RFLP, using *EcoR*I+*Sac*I and *Hind*III, as in Selosse et al. [5] was investigated to avoid repetitive sequencing of the same ITS. To investigate the phylogenetic position of the investigated orchid species, we amplified (conditions in Selosse et al. [5]) and sequenced (i) the plant ITS, using the plant-specific primer ITS1P; (ii) *rbcL *using primer rbcL1F (5'-ATGTCACCACAAACAGAAAC-3') and rbcL 1367R (5'-CTTCCAAATTTCACAAGCAGCA-3'); and (iii) *trnS-G *using a primer on *trnS *(5'-GCCGCTTTAGTCCACTCAGC-3') and the other on *trnG *(5'-GAACGAATCACACTTTTACCAC-3'). These loci were also amplified in other Neottieae, such as *Cephalanthera exigua*, *C. damasonium *Druce, *C. longifolia *(L.) Fritsch, *C. rubra *(L.) Rich., *Epipactis helleborine *(L.) Crantz, *E. muelleri *Godfery, *E. fageticola *(C.E.Hermos.) Devillers-Tersch. & Devillers, *E. fibri *Scappat. & Robatsch, *E. palustris *Crantz, *E. flava *Seidenf., *E. microphylla *Sieber. *ex *Nyman, *Neottia ovata *Bluff & Fingerh., *N. nidus-avis *(L.) Rich., *Limodorum abortivum *(L.) Sw., and *Thaia saprophytica *Seidenf. (Additional file 1). *Tropidia curculigoides *Lindl. was sequenced as an outgroup. Sequencing and sequence editing was performed as in Roy et al. [8] and corrected sequences (or consensus sequences for similar clones) were deposited in GenBank [75].

### Fungal identification and phylogenetic analyses

In order to identify fungi, a BLAST search for similar fungal sequences was conducted [76] using GenBank [75]. Two phylogenetic analyses were conducted, in order to (i) study the phylogenetic position of *Aphyllorchis *spp. using a concatenation of ITS, *rbcL *and *trnS-G *sequences, and (ii) to refine the phylogenetic positions of the many Russulaceae found in this study using ITS sequences (alignment and analysis were not possible for Thelephoraceae, because of too much variation in their ITS). Sequences of Neottieae and Russulaceae available in GenBank were downloaded and aligned together with ours using ClustalW [77], and then corrected by eye. Considering the high number of species of Russulaceae in GenBank, we used only species recorded from Thailand and species recovered when using BLAST for our sequences; the Russulaceae tree was not rooted. For Neottieae, *Tropidia polystachya, Nervilia shinensis *and *Vanilla planifolia *were chosen as outgroups. The phylogeny was computed by maximum likelihood with PhyML v2.4.4 [78]. For this analysis, a general time-reversible (GTR) model of DNA substitution was used [79,80], involving unequal base frequencies and six types of substitution. This model of DNA substitution was chosen using a series of hierarchical likelihood-ratio tests in Modeltest 3.7 [81]. Base frequencies were estimated, and 10,000 bootstrap replicates were performed. Phylogenetic trees were visualised using Figtree 1.1.2 [82].

### Isotopic sampling and analysis

Sampling for isotopic studies was conducted at three different sites (Doi Suthep #2, Doi Suthep #3 and Doi Pee Pan Nam; Table 1). At each site, we harvested *n *= 5 samples for aerial parts of MH orchids, leaves of four autotrophic species, and fruitbodies of up to six basidiomycetes species fruiting at sampling time (prioritising ECM species). All leaves were collected in close vicinity, at the same apparent light level and the same distance from the ground (less than 0.5 m) as orchids. When available, other terrestrial green orchids were collected (*Ludisia discolor *at Doi Suthep #3 and *Cheirostylis montana *at Doi Pee Pan Nam). Samples were dried at 65°C for 72 h and handled as in Tedersoo et al. [21] to measure total N, C/N ratio and abundances of ^13^C and ^15^N. Isotope abundances are expressed in δ^13^C and δ^15^N values in parts per thousand relative to international standards V-PDB and atmospheric N_2_: δ^13^C or δ^15^N = (R_sample_/R_standard _- 1) × 1000 (‰), where R is the molar ratio, that is, ^13^C/^12^C or ^15^N/^14^N. The standard deviation of the replicated standard samples (*n *= 13) was 0.031‰ for ^13^C and 0.237‰ for ^15^N. Total N, C/N ratio, δ^13^C and δ^15^N values were compared independently between species at each site by pairwise Mann-Whitney tests using Minitab™. Thus, groups of species were delimited for each variable and the Kruskal-Wallis test was performed, using these groups as a factor in order to study the validity of these groups more precisely.

### Fungal community analysis

To infer species from ITS sequences, we applied a threshold of 97.0% sequence identity over the whole ITS region; although there is no universally applicable threshold [83], this is in agreement with our previous studies [8,21]. Sequences were aligned using Bioedit and a similarity matrix was calculated. The frequency (p_i_) of each putative ECM species among individuals and within populations was calculated to establish a Shannon diversity index and a Simpson diversity index. Indices were compared between individuals by the pairwise Mann-Whitney test. To account for our variable sampling effort among orchid species, rarefaction curves were simulated 5,000 times using analytic rarefaction 1.3 [84] on two datasets: one pooling all populations for each species, and the other separating each population and calculating a mean value for each species. For a more qualitative analysis, fungal communities at the individual level were compared within and between species by building similarity matrixes with Primer 5.2.9 [85] using the Bray-Curtis similarity index. Two matrixes were computed by grouping fungal species into families (because no species or sequence was common between orchid populations or species, see below). DCA was performed with these matrixes, using population, forest type, geographical origin and species as factors.

## Abbreviations

AC: *Aphyllorchis caudata; *AM: *Aphyllorchis montana; *CE: *Cephalanthera exigua; *ECM: ectomycorrhiza or ectomycorrhizal; DCA: detrended component analysis; GTR: general time reversible (model); ITS: internal transcribed spacer of the ribosomal DNA; MH: mycoheterotroph.

## Authors' contributions

This article is part of MR's PhD thesis. M-AS and SV designed the research; SW and MR performed the sampling; MR and AS performed the molecular research; MR, M-AS and FR analysed the data; MR and M-AS wrote the paper. All authors have read and approved the final manuscript.

## Supplementary Material

Additional file 1 **Table S1**. Origin and accession numbers of orchids collected for the Neottieae phylogeny. Bold numbers indicate the sequences obtained in this study, the others were retrieved from GenBank. Click here for file

Additional file 2 **Table S2**. Fungi identified from *A. montana*, *A. caudata *and *C. exigua *mycorrhizae. ^α ^Putative species were delineated on a 97% internal transcribed spacer similarity threshold; whenever several species belong to the same taxon, their name includes a number to distinguish them, e.g. Russulaceae sp. #3. ^β ^Putative ecologies: ECM, ectomycorrhizal fungus; E: endophyte (*sensu *Julou et al. [41]); R: rhizoctonia; S: saprobe. ^γ ^ITS: Internal transcribed spacer; 28S: large ribosomal DNA subunit (28S); LrDNA: mitochondrial ribosomal DNA. Small letters refer to the DNA source used for identification, and upper numbers to the number of identical sequences retrieved from the same individual: d, direct PCR from root DNA; c, cloned PCR product from root DNA, r, inferred from RFLP profile; p, PCR from peloton DNA. Click here for file
